# Unraveling the Role of Alkali on Cobalt Catalyst Performance in Ethanol Steam Reforming by Operando DRIFT Studies and DFT Modeling

**DOI:** 10.1021/acsami.4c18402

**Published:** 2025-01-22

**Authors:** Gabriela Grzybek, Olga Wasiłek, Magdalena Greluk, Grzegorz Słowik, Arantxa Davó-Quiñonero, Agustín Bueno-López, Dolores Lozano-Castelló, Paweł Stelmachowski, Filip Zasada, Witold Piskorz, Andrzej Kotarba

**Affiliations:** † Faculty of Chemistry, Jagiellonian University in Krakow, Gronostajowa 2, 30-387 Krakow, Poland; ‡ Faculty of Chemistry, Maria Curie-Sklodowska University, Maria Curie-Sklodowska Sq. 3, 20-031 Lublin, Poland; § Department of Inorganic Chemistry, University of Alicante, Carretera de San Vicente s/n, E-03080 Alicante, Spain

**Keywords:** cobalt catalyst, ethanol steam reforming, alkali promotion, DFT modeling, operando DRIFT spectroscopy

## Abstract

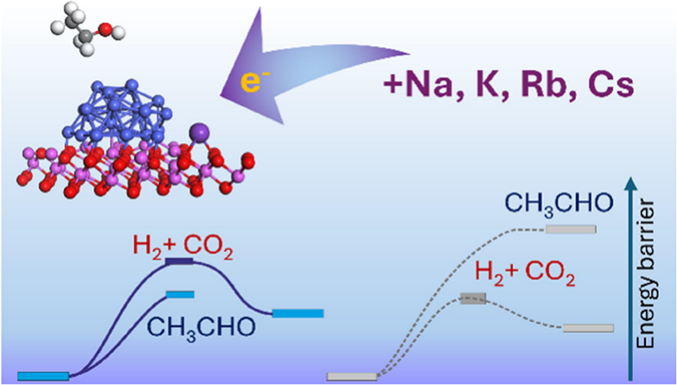

Hydrogen, a sustainable and environmentally friendly fuel, can be obtained through the ethanol steam reforming (ESR) process. The most promising catalysts for this process are those based on non-noble metals such as cobalt. The activity, selectivity, and stability of these catalysts strongly depend on the presence of alkali dopants. In this work, we have taken on the challenge of understanding the role of alkali. We synthesized a series of cobalt-containing catalysts supported on α-alumina and doped with Na, K, Rb, and Cs, which were thoroughly characterized using spectroscopic and microscopic techniques. We elucidated the significant difference in the efficiency of undoped and alkali-doped catalysts, based on diffuse reflectance infrared Fourier transform (DRIFT) operando spectroscopy studies under ESR conditions. The catalytic test results indicated a strong effect of alkali promoters on the interaction between the acetaldehyde byproduct and the Co/α-Al_2_O_3_ catalyst surface. Experimental data were confronted with the results of periodic DFT-GGA+U molecular modeling. It has been shown that electron transfer from alkali atoms to the cobalt active phase strongly influences the ethanol reforming pathway by increasing the adsorption energy of the aldehyde intermediate and facilitating the key C–C bond-breaking step.

## Introduction

The anticipated depletion of fossil fuel reserves is turning the world’s attention toward sustainable and environmentally friendly alternatives, such as using hydrogen as an energy carrier.^1,2^ Hydrogen is a clean-burning fuel, and when combined with oxygen in a fuel cell, hydrogen produces heat and electricity with only water as a byproduct, making it an emission-free solution. Among others, hydrogen can be produced by steam reforming using ethanol obtained from biomass, as described by eq 1:
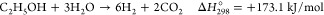
1In this process, many pathways lead to the formation of undesirable byproducts, which can adversely affect the performance of the catalyst and reduce the efficiency of the overall process.^3−6^ Ethanol under steam reforming process conditions can undergo the following transformations:^4^Dehydrogenation to acetaldehyde:
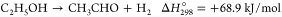
2Dehydration to ethylene:
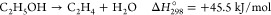
3Decomposition to methane, CO, and H_2_:
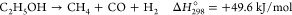
4Decomposition to acetone, CO, and H_2_:
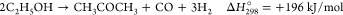
5Steam reforming to synthesis gas:
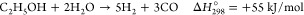
6In addition, each of the intermediate products can undergo the following reactions:Decomposition of acetaldehyde to methane and CO:
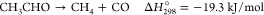
7Aldol condensation of acetaldehyde to acetone:
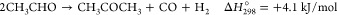
8Steam reforming of acetaldehyde to synthesis gas:
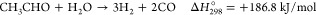
9Steam reforming of methane to synthesis gas:
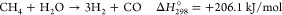
10Water gas shift reaction:
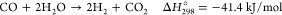
11Methanation reactions:
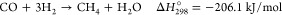
12
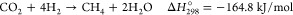
13Hence, there is a demand for the development of an active, selective, and stable catalyst capable of minimizing the contribution of these reactions in the steam reforming of ethanol. The supported catalysts containing noble metals (Rh, Ru, Pt, Ir) showed superior activity and stability.^7,8^ However, high cost limits their industrial application, and interest shifts to catalysts based on nonnoble metals such as Co, Ni, and Cu, of which cobalt-based catalysts seem to be the most promising systems.^2,4,9^

The metal active phases are most commonly supported onto the surface of metal oxides.^1,3,4,8,10^ Oxide supports have the advantage of storing oxygen on the surface, which limits the catalyst deactivation process. Surface oxygen reacts with undesirable byproducts of side reactions, reducing the formation of carbon deposits. The metal oxides most frequently mentioned in the literature as supports in ethanol steam reforming catalysts include oxides of aluminum, cerium, zinc, silicon, and magnesium.^1,4,11^ Among various polymorphs of alumina, α-Al_2_O_3_ possesses advantages such as being a cost-effective, readily accessible, and mechanically robust material, making it highly suitable for industrial applications.^12^ The most important advantage of this particular variant of alumina is its low acidity, which reduces the undesired pathways of the ESR process, especially ethanol dehydration.^1,12^

The performance of the ESR catalysts is significantly influenced by the alkali additives. They affect both the electronic properties of the active phase and the acidity of the support, thereby modifying the contribution of various reaction pathways of the ethanol steam reforming process. The positive effect of alkali promoters on the performance of ESR catalysts, frequently described in the literature, varies depending on the systems used and has been associated with the reduction of undesirable products such as C_2_H_4_ or CH_3_CHO, as well as the influence on the amount and degree of order of the resulting carbon deposits.^13−15^ In our previous studies, we demonstrated that in the case of a Co system on an α-Al_2_O_3_ support, the positive effect of potassium arises from the enhanced interaction between the cobalt phase and the support, which significantly increases the catalyst’s stability.^16,17^

Alkali-free alumina-supported cobalt catalysts have been investigated in theoretical studies.^18^ By cleaving the bulk structure of α-Al_2_O_3_ with various termination planes, it is feasible to create O-terminated, Al-terminated, and double Al-layer terminated α-Al_2_O_3_ (0001) surfaces, each possessing distinct characteristics.^19^ Among them, the nonpolar, reconstructed Al-terminated (0001) surface exposing 3-fold coordinated Al cations was found to be the most stable one in both experiment and simulation.^20,21^ DFT calculations for the Co_10_|α-Al_2_O_3_ (0001) surface suggest that C–C bond scission is favored at the Co^0^ site, while C–O bond scission is more likely to occur at the Co^x+^ site.^18^ These sites are also implicated in initiating the ESR reaction through the cleavage of the O–H bond in the ethanol molecule.

In this study, we aimed to elucidate the impact of various alkali promoters on the efficiency of the Co|α-Al_2_O_3_ catalyst, with a focus on explaining their role in the ESR process mechanism. The performance of catalysts doped with sodium, potassium, cesium, and rubidium was analyzed by considering the chemical characteristics of Co-active sites and the interactions between the catalysts and reaction intermediates. Operando DRIFT spectroscopy was utilized to gain insights into the mechanism of ethanol adsorption on the catalyst surface, as well as the formation of main and byproducts in the ESR process, both with and without alkali doping. The experimental findings were rationalized by results from periodic DFT-GGA+U molecular modeling using the VASP package. Slab models were developed, exposing the (0001) Al_2_O_3_ surface (Al_108_O_162_ stoichiometry) in contact with a cobalt cluster (Co_26_).

## Experimental Methods and DFT Modeling

### Materials

A series of alkali-doped alpha alumina-supported cobalt catalysts were prepared using the alumina support obtained from the New Chemical Synthesis Institute, INS (Puławy. Poland). About 8%wt. of cobalt spinel was deposited on the alumina supports by sonochemical method using Qsonica’s Q500 Sonicator ultrasonic probe to deposit the cobalt active phase on alumina support. The cobalt precursor Co(CH_3_COO)_2_·4H_2_O (Sigma-Aldrich) was mixed with about 90 mL of solvent (99.8% ethanol, Chempur), using a magnetic stirrer, and sonicated for 2 h (amplitude of 75%). Next, a 25% ammonia solution was added until a pH of 10.5 was reached and the sample was centrifuged and dried at 60 °C.

The alkali promoters in the amount of about 0.7 wt % were added to Co/α-Al_2_O_3_ catalyst by the incipient wetness impregnation method, using Na, K, Rb, or Cs nitrate aqueous solutions with appropriate concentrations. All alkali-doped catalysts were calcinated in air at 500 °C for 4 h.

### Characterization Methods

The composition of the catalysts was analyzed using an X-ray Fluorescence (XRF) spectrometer (Thermo Scientific Quant’X EDXRF Analyzer C10020) equipped with a 1 mm collimator and Mylar foil from Chemplex Industries, Inc. Phase composition was examined by X-ray diffraction (XRD) with a Rigaku Multiflex diffractometer, utilizing CuKα radiation (λ = 1.54 Å). Diffractograms were recorded over a 2θ range of 5–90°, with a step size of 0.02°. Additional structural analysis was performed using a Renishaw InVia Raman spectrometer (785 nm laser, range of 100–1000 cm^–1^). The reducibility of the samples was assessed via hydrogen temperature-programmed reduction (H_2_-TPR). For this, 50 mg of the sample was placed in a fixed-bed quartz flow microreactor system, coupled to a thermal conductivity detector (TCD3, Valco, Houston, TX, USA), and reduced in a 5 mol % H_2_ in Ar gas mixture (99.999% purity) with a flow rate of 10 mL·min^–1^, across a temperature range of 80–950 °C (heating rate of 10 °C·min^–1^). A cold trap was employed to remove water during the measurements.

Microscopic analysis of both fresh and spent catalysts was conducted using a scanning transmission electron microscope (TEM, Titan G2 60–300 kV, FEI Company) operating at an electron beam acceleration voltage of 300 kV. Phase separation was achieved through fast Fourier transform (FFT) utilizing masking features available in the Gatan Digital Micrograph software package. Previous studies provide further details of the measurement procedures.^22^

X-ray photoelectron spectroscopy (XPS) studies were performed using a multichamber ultrahigh vacuum system (PREVAC), equipped with a SESR4000 electron analyzer. The system included a Al Kα source operating at 250 W at 1486.6 eV emission energy. For survey spectra, the analyzer’s pass energy was set at 200 eV with a step size of 500 meV, while high-resolution spectra (Co 2p, O 1s, Na 1s, K 2p, Rb 3d, Cs 3d) were recorded with a pass energy of 50 eV and step sizes of 50–100 meV. The base pressure in the analysis chamber was 5 × 10^–9^ mbar, and during spectra acquisition, the pressure remained below 3 × 10^–8^ mbar. Data analysis was performed using CasaXPS software (v 2.3.23 PR1.0).^23^

### Catalytic Tests

Catalytic experiments were conducted in a quartz reactor under atmospheric pressure, using an ethanol-to-water molar ratio of 1:4 and a reaction temperature of 500 °C. The catalyst bed consisted of 0.1 g of catalyst with a particle size between 150 and 300 μm, mixed with quartz scraps. Prior to the reaction, the samples were reduced in a hydrogen flow at 550 °C for 1 h. Reaction products were analyzed using two gas chromatographs: a Bruker 450-GC equipped with Porapak Q and CP-Molsieve 5 Å columns, and a Bruker 430-GC equipped with a Molsieve 5 Å column.

The conversion of ethanol (*X*_EtOH_) and conversions of ethanol into individual carbon-containing products (*X*_CP_) were calculated based on its concentrations:
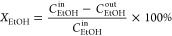
14
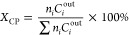
15where  is the molar concentration of ethanol in the reaction mixture (mol %);  is the molar concentration of ethanol in the postreaction mixture (mol %);  is the molar concentration of carbon-containing product in the postreaction mixture (mol %); *n_i_* is the number of carbon atoms in a carbon-containing molecule of the reaction product.

The selectivity of hydrogen formation was determined as follows:
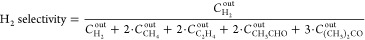
16where *C*^out^ is the molar concentration of the hydrogen-containing products in the postreaction mixture (mol %).

### Operando DRIFT Studies under Conditions of the Ethanol Steam Reforming

The spectroscopic measurements were performed using the PerkinElmer Spectrum 3 Tri-Range MIR/NIR/FIR Spectrometer coupled with the Pfeiffer Vacuum Prisma PRO mass spectrometer. The sorption of substrates and reaction products of steam reforming of ethanol on the surface of studied materials was investigated. A mixture of water and ethanol (molar ratio H_2_O:EtOH was 4:1) was introduced into the system using an INSTECH peristaltic pump (model P720) at a rate of 0.01 mL/min to the vaporizer, which heated the injected mixture. The vapors were introduced into the measuring chamber at a helium flow rate of 70 mL/min, which was used as the carrier gas.

The measurements were conducted in the temperature range from 200 to 500 °C. Spectra were collected for each sample every 5 min at a given temperature under the conditions of the ethanol steam reforming reaction. At the same time, mass spectra for main products and byproducts were collected in real time during the measurement. Studies of ethanol absorption on the surface of the catalysts were also conducted.

### Parametrization of DFT Calculations

For molecular modeling, we utilized a periodic, spin-unrestricted DFT+U approach with the PW9^24^ exchange-functional, implemented within the projector augmented plane wave method available in the VASP code.^25^ A Hubbard parameter (U) value of 3.5 eV was chosen for Co atoms to capture the Coulomb on-site repulsion in a highly correlated system. Brillouin zone sampling was achieved using a standard Monkhorst–Pack grid^26^ with a 5 × 5 × 5 mesh for bulk calculations and a 5 × 5 × 1 mesh for slab calculations. A cutoff energy of 500 eV and an SCF convergence criterion of 1 × 10^–6^ eV were employed. Atomic charges were determined using Bader population analysis.^27^ The treatment of dispersion forces was carried out using the Tkatchenko and Scheffler method (DFT-TS).^28^ To compute the transition states of key steps of the ESR mechanism we utilized the nudged elastic band method with Climbing Image correction (cNEB).^29,30^ For each TS search, five to nine NEB images encompassing the initial and final states were employed.

### Slab Model Construction

The surface supercell (3 × 3 × 1) slab model of the α-Al_2_O_3_ plane was constructed by cleaving the bulk corundum crystal (introducing a vacuum region of ∼15 Å to avoid unphysical interactions) using the unit cell parameters obtained from experimental data and then optimized with the employment of Birch–Murnaghan equation^31^ (*a* = *b* = 4.754 Å, *c* = 12.982 Å, α = β = 90°, γ = 120°). For exposed termination, we chose the nonpolar Al-terminated (0001) surface exposing 3-fold coordinated Al cations (see Figure S1a), and the proposed stoichiometric Al_108_O_162_ slab consisted of an 11 Å thick oxide layer (equivalent to 16 atomic layers). Such an initial slab model was optimized (relaxation was limited to the top four atomic layers). As a result of relaxation, a noticeable decrease in the *z*-position of exposed aluminum atoms is observed (which is associated with the shortening of surface O–Al bonds) leading to the leveling of the aluminum and oxygen layers. The final geometry (Figure S1b) was found to be similar to that described previously for α-Al_2_O_3_(0001) as the most stable one in both experiment and simulation.^20,21,32^

## Results and Discussion

To understand the nature of the alkali promotion effect on the cobalt catalyst efficiency in the ethanol steam reforming process, we have thoroughly investigated a series of Na, K, Rb, and Cs-doped Co-containing alumina-supported catalysts. We have used pure alpha alumina polymorph in order to eliminate the ethanol dehydration path facilitated when acidic support is used.^12^ The cobalt active phase, in the amount of about 8 wt %, was introduced onto the support surface using the sonochemical synthesis method that had been optimized previously.^12^ For all alkali-promoted catalysts approximately 0.7 wt % of alkali was introduced by impregnation method.^17^Table 1 summarizes the results of elemental analysis for the series of alkali-doped Co/α-Al_2_O_3_ catalysts.

**Table 1:** Bulk and Surface Content of Cobalt and Alkali Metals in Synthesized Samples Determined Using the XRF and XPS Methods

	bulk content (wt %)		surface content (atom %)	
sample	Co	alkali	Co	alkali
Co/α-Al_2_O_3_	8.5		13.3	
Na_Co/α-Al_2_O_3_	8.2	0.7	4.7	8.9
K_Co/α-Al_2_O_3_	8.5	0.7	5.6	3.6
Rb_Co/α-Al_2_O_3_	7.7	0.8	5.7	2.1
Cs_Co/α-Al_2_O_3_	7.8	0.7	4.9	3.4

**Figure 1:**
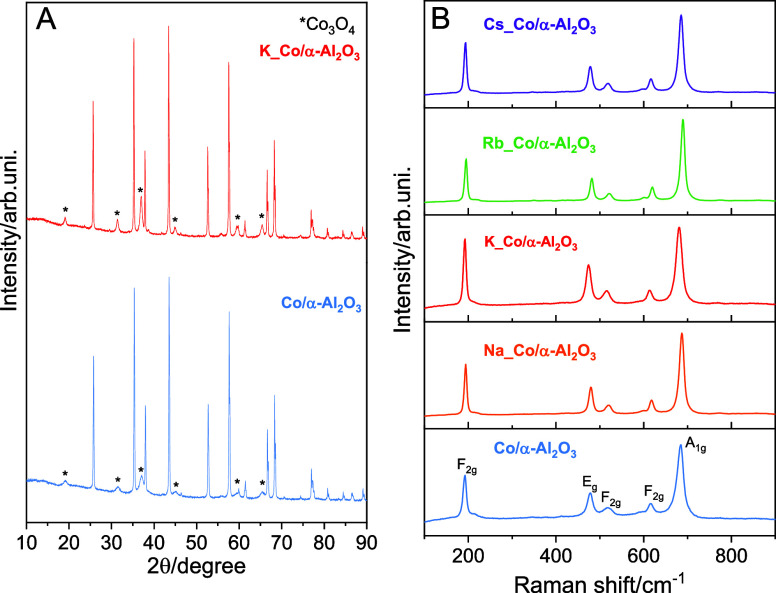
(A) XRD patterns of the Co/α-Al_2_O_3_ and K_Co/α-Al_2_O_3_ catalysts and (B) Raman spectra for a series of catalysts.

The presence of the cobalt phase, as well as alkali promoters in the systems, was confirmed using X-ray photoelectron spectroscopy (XPS, Figures S3 and S4). A narrow-scan analysis of the regions allowed for defining their surface concentration (Table 1). The results of the bulk and surface composition indicate that the surface of alkali-doped catalysts is enriched with alkali species and depleted from cobalt compared to the bulk. The phase composition of the investigated catalytic systems was determined through X-ray diffraction (XRD) and Raman spectroscopy (Figure 1). The diffractograms for both Co/α-Al_2_O_3_ and K_Co/α-Al_2_O_3_ are dominated by high-intensity peaks characteristic of the α-phase of aluminum oxide (α-Al_2_O_3_).^33^ Reflections indicated with (*) at 2θ values of 19°, 33°, 36°, 60°, and 65° correspond to the (111), (220), (311), (511), and (440) planes of Co_3_O_4_ (ICSD-69378), respectively. It is important to note that spinel-related reflections in the XRD patterns may also arise from Co–Al mixed spinel. As anticipated, the small amount of alkali promoter used did not cause any significant differences in the XRD pattern (as presented in the representative diffractogram for K_Co/α-Al_2_O_3_).

The spinel structure of the cobalt active phase precursor was confirmed by the Raman spectroscopy. The spectra of each sample display characteristic bands at 191, 479, 515, 616, and 684 cm^–1^, with symmetries of F_2g_, E_g_, F_2g_, F_2g_, and A_1g_, respectively, indicating the presence of the spinel phase (Figure 1B). In addition, the asymmetry of the A_1g_ peak suggests that the cobalt spinel phase is accompanied by a small share of the mixed cobalt-alumina spinel phase, as we have shown in our previous papers.^12,34^ No significant differences are observed in the Raman spectra recorded for the Co/α-Al_2_O_3_ sample, and the systems doped with Na, K, Rb, and Cs, indicating that the addition of alkali promoters does not affect the coordination environment of cobalt ions. The broad maxima in the H_2_-TPR profiles (see Figure S5) confirm the share of the CoAl_2_O_4_ phase. As Co^2+^ in a mixed Co–Al oxide matrix is more difficult to reduce, it needs a much higher temperature to reduce Co^2+^ to Co^0^ than in a Co_3_O_4_ matrix.^12,33^

**Figure 2:**
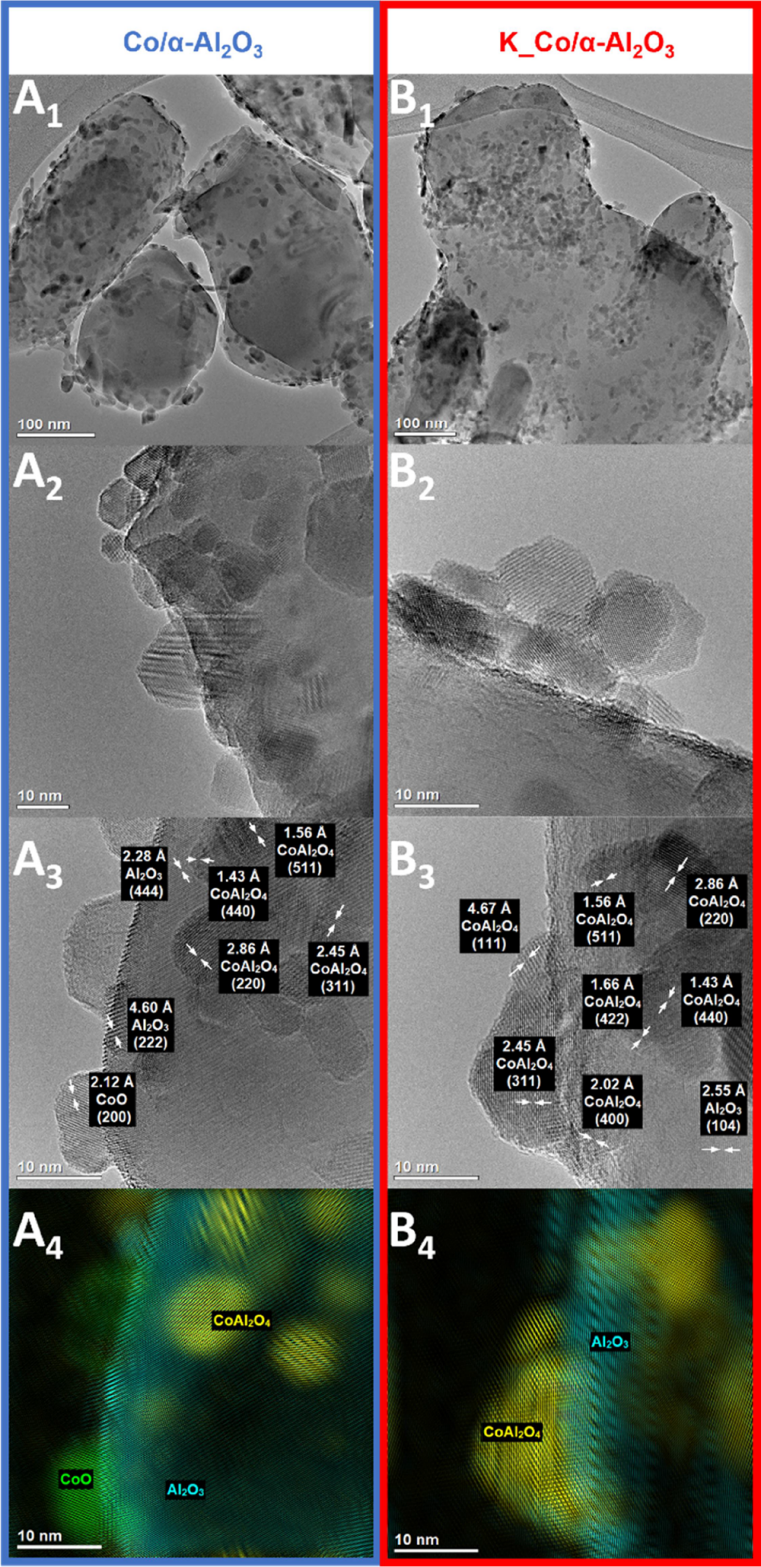
Microscopic analysis of the Co/α-Al_2_O_3_ and K_Co/α-Al_2_O_3_ catalysts: TEM images (A_1_,A_2_,B_1_,B_2_) with phase analysis (A_3_,A_4_,B_3_,B_4_).

Figure 2 displays the microscopic analysis of the Co/α-Al_2_O_3_ catalyst and K_Co/α-Al_2_O_3_ as a representative of the alkali-doped catalysts. From these studies, the size of the α-Al_2_O_3_ support crystallites was determined to be several hundred nanometers (Figure 2A_1_,B_1_). A fairly uniform dispersion of the cobalt phase crystallites, observed for both samples, results from using the optimized sonochemical deposition. The phase identification confirmed the presence of the mixed cobalt–aluminum spinel CoAl_2_O_4_ (see details in SI, Figure S6), with an average size of about 10 nm. The presence of the Co–Al mixed oxide, surprising due to the very low reactivity of the α-Al_2_O_3_, we explained in our previous paper by the extremely high local temperatures and pressures (∼5000 K, ∼1000 bar) accompanying the sonochemical synthesis.^12^ In the case of the undoped sample, additionally small CoO crystallites were observed (Figure 2A_3_,A_4_).

**Figure 3:**
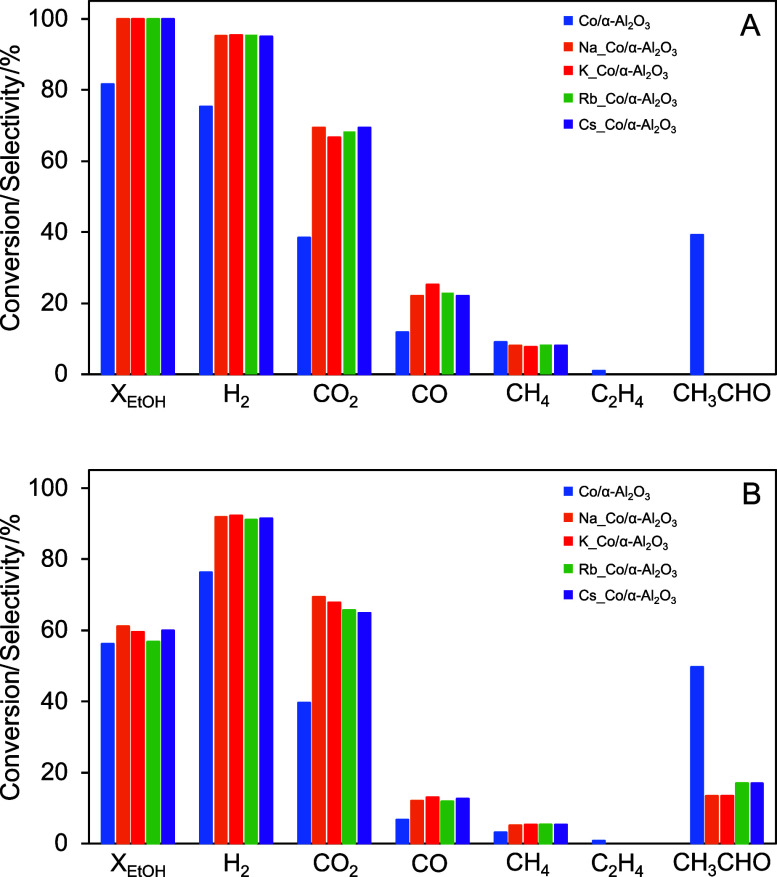
Ethanol conversion and selectivity to individual products at the beginning (A) and after 21 h (B) of the ESR process (500 °C, EtOH/H_2_O = 1:4) over undoped-, and alkali-doped catalysts.

Prior to the catalytic tests, the samples were reduced and tested in the steam reforming process in the isothermal mode at a temperature of 500 °C using a reaction mixture with ethanol to water molar ratio 1:4. Figure 3 shows the results of ethanol conversion and selectivity to individual reaction products at the beginning (A) and after 21 h (B) of the ESR process (the complete catalytic test runs are presented in SI, Figure S7). In the applied reaction conditions, the Co/α-Al_2_O_3_ catalyst maintains a stable ethanol conversion rate of approximately 60%. For this alkali-free sample, the selectivity toward the main steam reforming products is the lowest, with about 75% for H_2_ and 40% for CO_2_. The undoped catalyst also exhibits the lowest selectivity to CO at around 7% and to CH_4_ at about 3% while showing the highest selectivity to the undesired byproducts, such as CH_3_CHO at roughly 50% and C_2_H_4_ at around 1.2%. The minimal production of ethylene, and thus the suppression of the ethanol dehydration pathway, is attributed to the low acidity of the α-Al_2_O_3_ carrier surface. Catalysts doped with sodium, potassium, rubidium, and cesium initially exhibit higher ethanol conversion, which gradually decreases over time, eventually stabilizing around 60%, similar to the Co/α-Al_2_O_3_ catalyst. The significant drop in conversion for the alkali-doped systems was previously explained in our work by the initial migration of alkali promoters.^17^ The selectivity of the alkali-doped systems to H_2_ and CO_2_ is much higher compared to the alkali-free sample, reaching approximately 90 and 70%, respectively. A similar trend is observed for CO and CH_4_ selectivity. Conversely, the selectivity to the undesired products is much lower than that of the Co/α-Al_2_O_3_ catalyst. It is important to note that the selectivity to certain reaction products changes over the 21 h duration of the ESR process (see comparison of Figure 3A,B). The most significant change concerns selectivity toward acetaldehyde. Initially, it is not detected in the reaction products over the alkali-doped catalysts. However, after 21 h of the process it appears with selectivity reaching 15–20%. Despite this increase, the selectivity remains significantly lower compared to that of the nondoped catalyst. The appearance of aldehyde after 21 h of the ESR process is the result of the catalyst’s gradual deactivation during the ESR process, primarily due to carbon deposition. This carbon deposit blocks active reaction sites, altering the contributions of individual reaction pathways over time and affecting product selectivity.

The Co/α-Al_2_O_3_ and K_Co/α-Al_2_O_3_ catalysts were analyzed after 21 h of ESR process to assess the carbon deposits formed on the catalyst beds. Transmission electron microscopy (TEM) was used to examine the type and location of the deposits. As shown in Figure S8, a considerable amount of carbon deposition occurred on both catalysts during the steam reforming process. For the undoped catalyst, the carbon deposit is observed to largely separate the cobalt phase from the support. In contrast, in the K-doped catalyst, this separation was significantly reduced, with the cobalt nanocrystals remaining in direct contact with the alumina support. These findings align with previously reported substantial improvement in the interaction between the active phase and alumina support due to the presence of potassium.^16,17^

**Figure 4:**
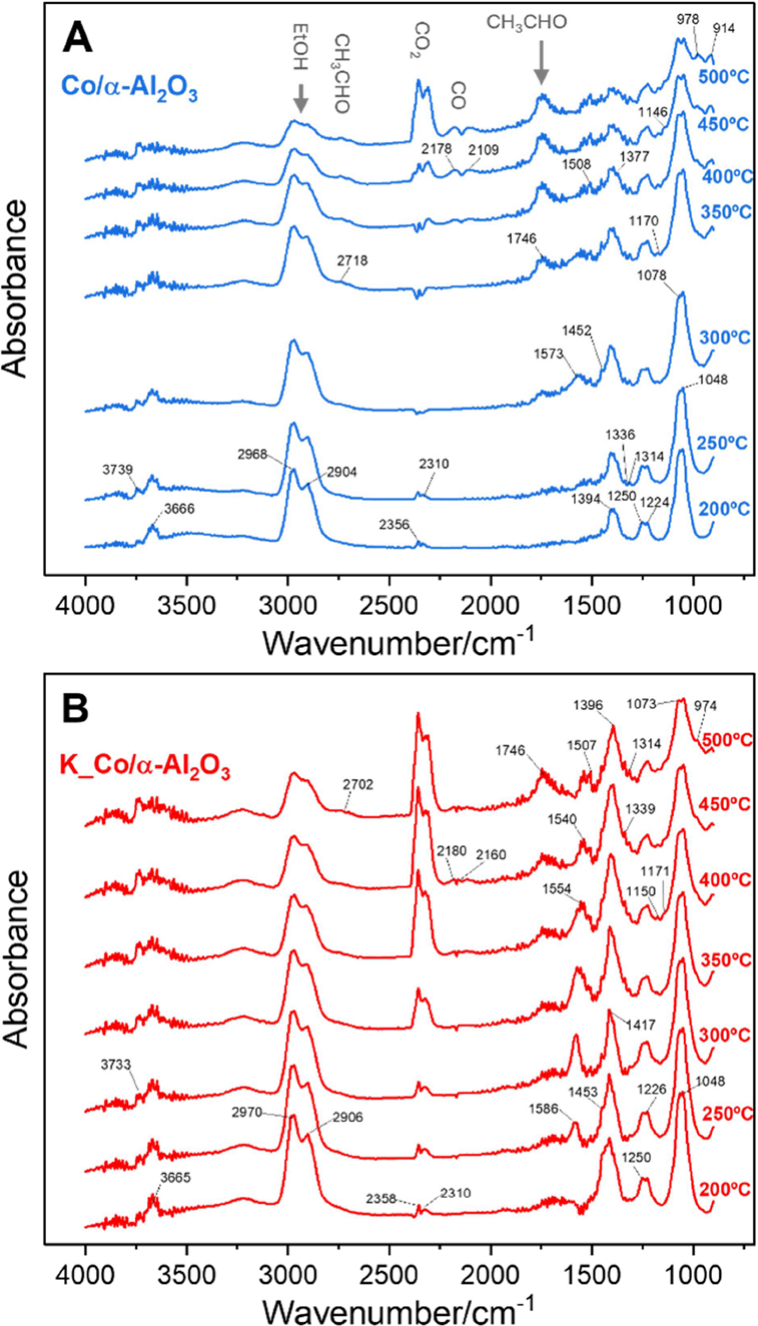
DRIFT spectra of Co/α-Al_2_O_3_ (A) and K_Co/α-Al_2_O_3_ (B) catalysts under EtOH:H_2_O mixture conditions (1:4) and at various temperatures.

To elucidate better the mechanism of the catalytic process, we performed *operando* DRIFT studies under conditions of ethanol steam reforming in the temperature range from 200 to 500 °C. In Figure 4, the results for undoped and potassium-doped catalysts are compared (the results for the rest of the alkali-doped catalysts are presented in Figure S9). The bands at 3739 and 3666 cm^–1^ are due to vibrations of the O–H bond (ν(OH)), corresponding to hydroxyl groups and the water adsorbed on the catalyst surface (Table S2). These bands are present in both undoped and alkali-doped catalysts (for this sample at 3733 and 3665 cm^–1^, respectively). The bands at 2968, 2904, 1078, and 1048 cm^–1^, which appear as early as 200 °C, correspond to the vibrations of ν_as_(CH_3_), ν_s_(CH_3_), and ν(CO).^35−37^ The bands characteristic for acetate groups are visible on the spectra of the Co/α-Al_2_O_3_ catalyst: 1573, 1508, and 1452 cm^–1^, attributed to symmetric and asymmetric vibrations ν_as_(OCO), δ_as_(CH_3_), and ν_s_(OCO), respectively. These broad bands are attributed to the formation of ethoxy groups, indicating the dissociative adsorption of ethanol on the catalyst surface. As the temperature increases above 300 °C, the intensity of these bands decreases, suggesting a lower concentration of ethanol, likely due to bond breaking and the formation of reaction products on the catalyst surface.

For the Co/α-Al_2_O_3_ catalyst, at temperatures above 350 °C, an increase in the intensity of the bands corresponding to carbon oxides is observed: 2356 and 2310 cm^–1^ for gas phase CO_2_, along with the emergence of bands at 2178 and 2109 cm^–1^ for CO. The increased intensity of the CO_2_ bands indicates high catalyst activity at temperatures above 350 °C. For alkali-doped catalysts, these products are observed at much lower temperatures. For example, in the presence of a catalyst containing potassium or rubidium, these products appear at temperatures as low as 200–250 °C (Figure S7). Moreover, in DRIFT spectra, signals corresponding to surface groups formed as a result of the oxidation of ethoxy groups are visible. The band at 1746 cm^–1^ of C=O vibrations, along with the presence of bands at 2718 and 978 cm^–1^, appears at temperatures higher than 300 °C, indicating the presence of acetaldehyde in the system. These bands increase with rising temperature up to 300 °C and disappear above this threshold value. The appearance of these vibrations suggests the oxidation of ethoxy groups to acetaldehyde, and subsequently to the acetyl form. In the 1600–900 cm^–1^ zone, bands from the surface carbonates are visible, with the intense band at 1394 cm^–1^ coming from polydentate carbonate species.^38^ The spectra for alkali-doped catalysts show important differences from those of the Co/α-Al_2_O_3_ sample. In the case of the K_Co/α-Al_2_O_3_, the bands due to CO_2_ are much more intense than for the undoped sample, but there is a noticeable decrease in the intensity of the bands around 2100–2200 cm^–1^ coming from CO. It is worth noting that for the K_Co/α-Al_2_O_3_ the aldehyde-derived bands appear at much higher temperatures (400–450 °C) and their intensity relative to the CO_2_-derived bands is much lower. This indicates the lower CH_3_CHO production in the system. The lower CH_3_CHO production for the K_Co/α-Al_2_O_3_ was also confirmed in the catalytic test results presented above (Figure 3).

**Figure 5:**
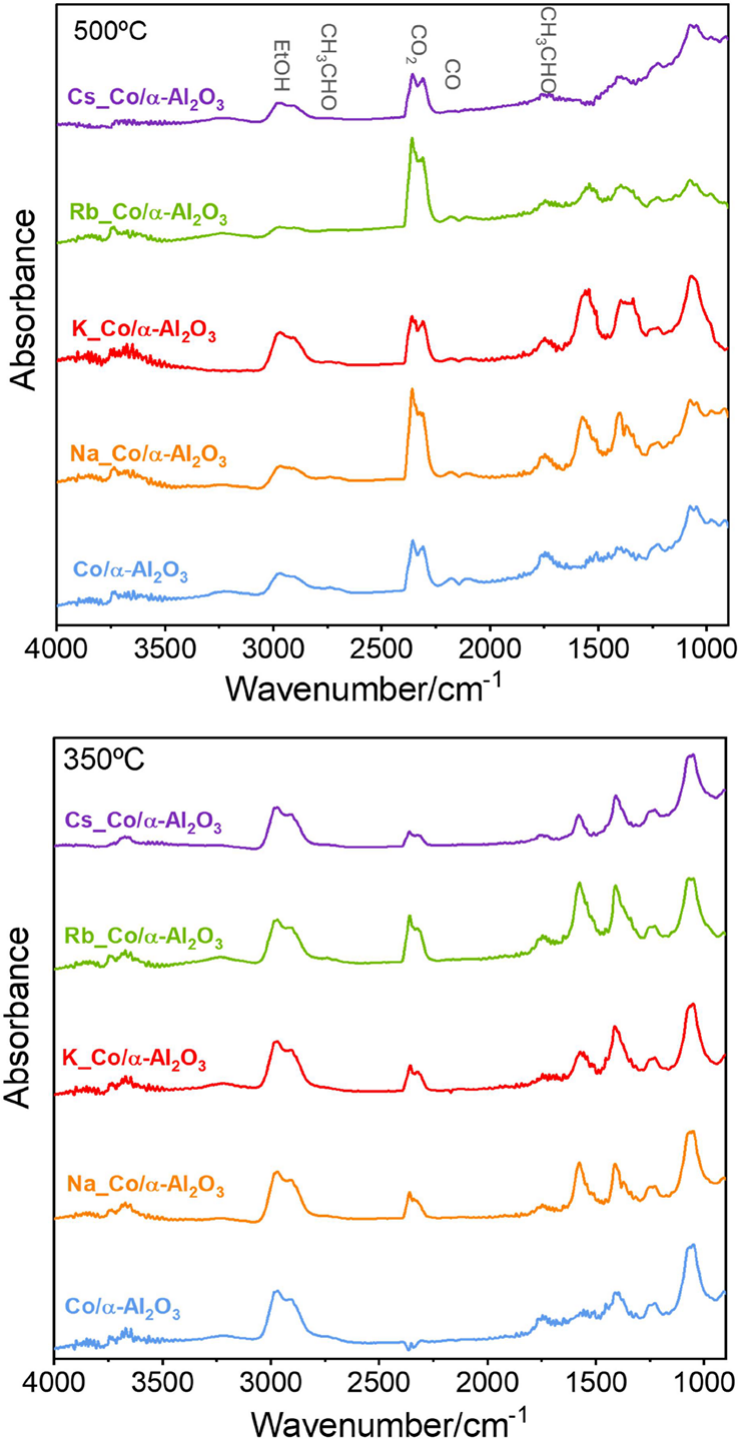
DRIFT spectra of all studied catalysts collected at 350 and 500 °C under EtOH:H_2_O mixture conditions (1:4).

A comparison of the results for all the investigated alkali promotes shows that the beneficial effects are very similar (Figure S7). For all of them, the bands due to CO_2_ are much more intense at lower temperatures. Thus, it is clear that for alkali-doped catalysts, the steam-reforming reaction of ethanol starts at lower temperatures. Figure 5 illustrates how the reaction’s progression is affected by temperature. At 500 °C, the ethanol bands are less intense compared to those at 350 °C, while the product bands become more prominent, indicating substantial ethanol transformation.

To elucidate the significant influence of alkali addition on selectivity toward CH_3_CHO and to test the hypothesis that the alkali promotion effect is of an electronic nature, we conducted periodic DFT-GGA+U molecular modeling (for its parametrization see SI, Chapter 3.1). The optimal geometries of the cobalt cluster stabilized on the alumina surface, both with and without potassium doping (referred to as Co_26_|α-Al_108_O_162_(0001) and e-K-Co_26_|α-Al_108_O_162_(0001), respectively), are detailed in the SI (see Supporting Information, Figures S1 and S2). The partial charges for the Co atoms are provided in Table S1.

The charge distribution analysis reveals that in the case of the undoped Co_26_|α-Al_108_O_162_(0001) catalyst, the overall electron transfer is minimal (Δ*q*_B_ = −0.08 e), with the charge evenly distributed across the cobalt cluster atoms. However, in the K-doped catalyst, the potassium atom donates an electron density (becoming a K^+^ cation with *q*_B_(K) = 0.98 |e|), which is localized almost entirely on the cobalt phase. A detailed examination of the charge distribution within the Co_26_ cluster shows that the upper layer retains a nearly metallic character (Co^0^), while the deeper atoms acquire a negative charge, suggesting partial reduction. A similar effect (of negatively charged TMI cluster over potassium-doped Ni|Al_2_O_3_ catalyst) was recently reported by Wang and co-workers.^39^

**Figure 6:**
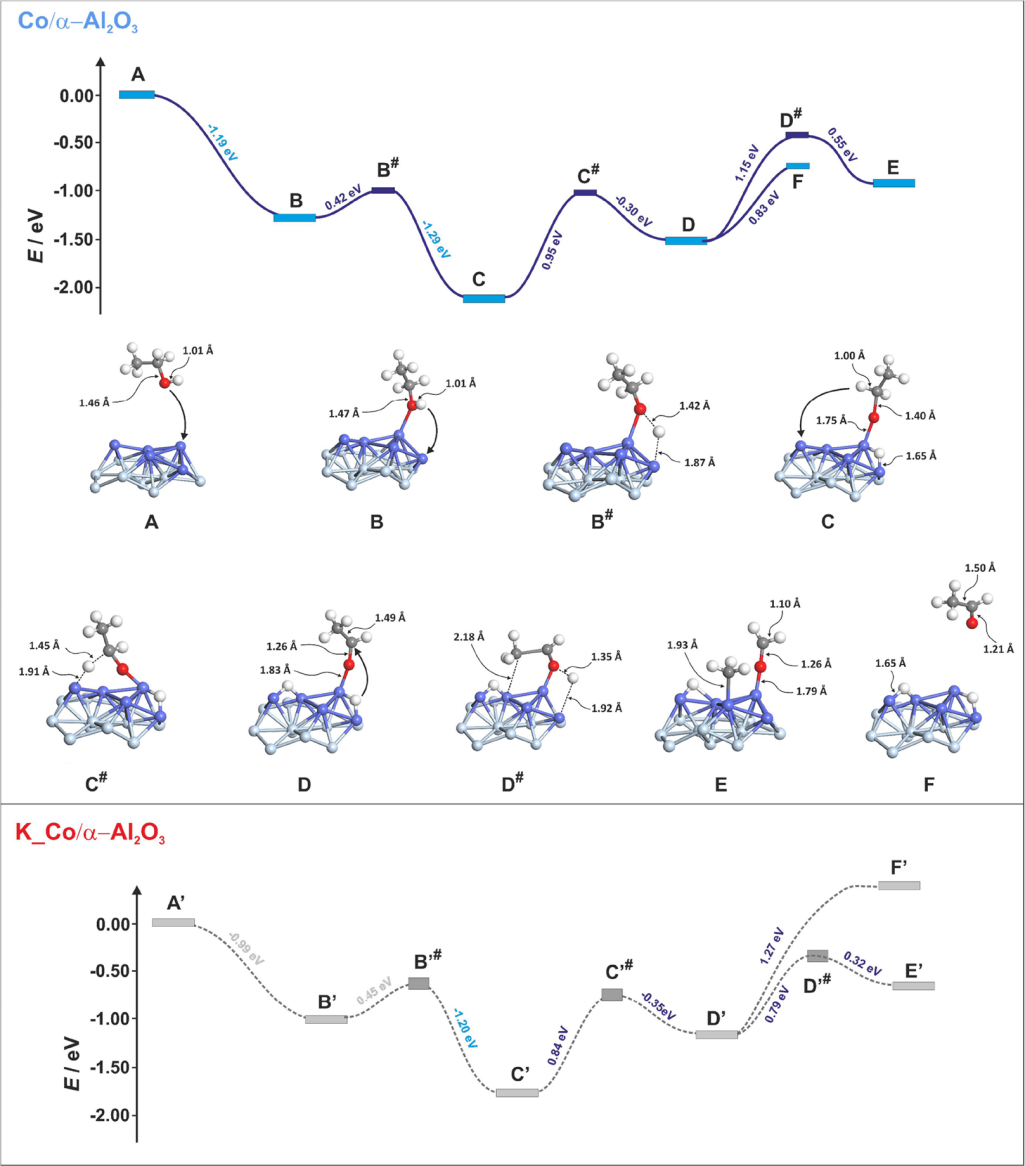
Energy profile of the key steps of the ESR reaction running on a bare Co/α-Al_2_O_3_ catalyst (top panel) together with geometries of corresponding intermediates and transition states and the comparative profile calculated for the potassium-doped system (bottom panel).

Literature on molecular modeling of ethanol reforming on Co-containing catalysts highlights various possibilities for molecule adsorption and activation, proposing multiple parallel pathways leading to final products (CO_2_ + H_2_O) as well as undesirable byproducts like formaldehyde or carbonization products.^18,40−43^ Previous computational studies suggest that the reaction preferably occurs through surface activation and double dehydrogenation of the CH_3_CH_2_OH molecule, leading to a transient surface-stabilized acetaldehyde intermediate.^18,40,41^ The cleavage of the C–C bond in the aldehyde is identified as a critical step in driving the overall reaction, and the competitive desorption of this intermediate affects the experimentally observed selectivity of the catalytic process. Experimental evidence suggests that these critical steps are strongly influenced by the presence of alkali on the catalyst surface (see Figure 3). Consequently, we compared the molecular modeling of these steps for the Co_26_|α-Al_108_O_162_(0001) catalyst and its potassium-modified counterpart (K–Co_26_|α-Al_108_O_162_(0001)). In the case of the undoped catalyst the key steps of ESR mechanism are presented as an energy profile in the top panel of Figure 6, together with corresponding epitomes showing the geometries of intermediates and transition states (marked with # signs). In the initial stage, gaseous ethanol (**A**) adsorbs onto the top cobalt center of the Co_26_ cluster through its oxygen atom (**B**). This adsorption process occurs without an activation barrier, resulting in a stabilization energy of Δ*E*_rnx_ = −1.19 eV. In the next step adsorbed molecule undergoes dehydrogenation (involving the proton of the OH group), leading to the formation of a surface-bound ethoxy group (**C**). The activation barrier for this step, associated with transition state **B**^**#**^, is Δ*E*_a_ = 0.42 eV, and the system undergoes further stabilization (Δ*E*_rnx_ = −0.87 eV). The subsequent dehydrogenation step (**C → C**^**#**^**→ D**) involves the α-carbon and is significantly more challenging, with an activation energy of Δ*E*_a_ = 0.95 eV. This process is endothermic (Δ*E*_rnx_ = 0.60 eV), resulting in the formation of surface-stabilized acetic aldehyde. The next key step is the C–C bond breaking, which preferably occurs simultaneously with the reattachment of the previously detached hydrogen (**D → D**^**#**^**→ E**). In this mechanism, the aldehyde tilts toward the surface, forming a Co-CH_3_ bond (see TS geometry **D**^**#**^), and one hydrogen is transported to the carbonyl carbon. This rearrangement provides a lower activation barrier (Δ*E*_a_ = 1.15 eV) compared to direct bond cleavage, following the mechanism proposed by Balbuena et al.,^41^ with Δ*E*_rxn_ for this step equaling 0.60 eV. A critical aspect for the selectivity of the ESR process is the potential desorption of the intermediate aldehyde from the surface. This is illustrated in step **D → F**, where desorption from the unpromoted catalyst requires only 0.83 eV of activation energy. This relative ratio of calculated barrier heights indicates that the aldehyde’s desorption process can be efficient, significantly reducing the reaction’s selectivity. For the potassium-promoted catalyst, the energy profile corresponding to the analogous reaction steps is depicted in the bottom panel of Figure 6 (with intermediates and transition states denoted by an apostrophe). A comparison with the profile of the undoped catalyst reveals that the initial stages—ethanol adsorption and its first and second deprotonation—proceed similarly. The key difference in the energy profile emerges in the final stages. Unlike the undoped catalyst, the direct desorption of the intermediate aldehyde product requires a higher activation energy (1.27 eV) than the C–C bond cleavage (0.79 eV). This reversal of barrier heights for these critical steps clearly indicates that with potassium doping, the more strongly stabilized aldehyde is more likely to undergo fragmentation via C–C bond cleavage, which experimentally correlates with increased activity and selectivity in the ESR process. The observed difference in energy profiles may be related to the electronic effect resulting from the flow of charge density onto the cobalt cluster (see Table S1 in SI). The additional electron causes the center of gravity of the d-band of cobalt to shift toward the Fermi level (similar to the effect described by Wang et al.^39^ and favors the transfer of an electron to the intermediate aldehyde product. In turn, the additional charge accumulated in adCH_3_COH upon potassium doping (calculated as Δ*q*_B_ = −0.21 |e|) causes a change in the strength of the important bonds of the aldehyde intermediate. Indeed, the analysis of the key bond orders indicates that the internal C–C bond weakens under the influence of potassium (Meyer bond order decrease from 1.25 to 1.09), whereas the Co–O bond, responsible for anchoring the adCH_3_COH to the surface, strengthens (from 0.69 to 0.86 Mayer unit).

The activation barriers for key steps of the ESR reaction were also calculated with different alkali promoters (Na, Rb, Cs) and the results of these simulations are summarized in Table 2. It can be found, that the effect of other alkaline promoters is analogous to the effect of potassium. The barrier for C–C bond cleavage is significantly lowered in each case (by 0.25 eV on average), and the stabilization of the acetaldehyde intermediate is increased (by ∼0.28 eV on average) regardless of the alkaline dopant used. These values correlate with corresponding C–C and Co–O bond orders (see Table 2), which indicate that the catalyst behavior is influenced mainly by electron factors resulting from the electron density promotion of the cobalt cluster provided by alkaline dopants.

**Table 2:** DFT Calculated Activation Barriers (Δ*E*_act_) of Key Steps of the ESR Process over Bare and Alkali-Doped Catalysts, and Important Bond Orders within the adCH_3_CHO Intermediate Product

	Δ*E*_act_ (eV)		Mayer bond order	
catalyst	C–C cleavage	CH_3_CHO desorption	C–C	Co–O
Co_26_|α-Al_108_O_162_	1.15	0.83	1.25	0.69
Na|Co_26_|α-Al_108_O_162_	0.85	1.14	1.08	0.82
K|Co_26_|α-Al_108_O_162_	0.79	1.27	1.09	0.86
Rb|Co_26_|α-Al_108_O_162_	0.82	1.23	1.11	0.87
Cs|Co_26_|α-Al_108_O_162_	0.89	1.09	1.07	0.84

## Conclusions

The positive effect of alkali doping on the performance of the Co/α-Al_2_O_3_ catalyst in the steam reforming of ethanol is reflected in increased production of the desired reaction products, H_2_ and CO_2_, by changing the reaction pathway. The catalytic results were supported by diffuse reflectance infrared Fourier transform (DRIFT) operando spectroscopy studies, which showed a higher contribution of CO_2_ over CH_3_CHO in the products of the ESR process on the alkali-doped catalyst surface. Thus, the presence of alkali promoters favors the cleavage of the C–C bond in the intermediate product, CH_3_CHO, and the resulting formation of CO_2_. This effect was consistently observed regardless of the type of alkali promoter (Na, K, Rb, or Cs) present on the catalyst surface. To better understand the nature of this effect, detailed DFT studies were conducted on the K-Co_26_|α-Al_108_O_162_(0001) system, revealing that the alkali promoter transfers electrons to neighboring Co_26_ atoms, increasing the electron density at the active Co sites, which in turn leads to stronger adsorption of the formaldehyde intermediate, and facilitate the key C–C bond-breaking step and enhancing hydrogen selectivity.

## Supporting Information Available

Detailed description of the DFT studies and the additional figures presenting XPS spectra, H_2_-TPR profiles, FFT patterns, catalytic test results, microscopic analysis of the spent catalysts, and DRIFT spectra (PDF)
