# Supplementary material for: Unraveling the Role of Alkali on Cobalt Catalyst Performance in Ethanol Steam Reforming by Operando DRIFT Studies and DFT Modeling
Source: ACS Appl Mater Interfaces. 2025 Jan 22;17(5):7697–706. doi: 10.1021/acsami.4c18402 (PMC11803558; doi:10.1021/acsami.4c18402)
Supplement: Supplementary file 1 — am4c18402_si_001.pdf [file am4c18402_si_001.pdf]

Supporting Information

for

Unraveling the Role of Alkali on Cobalt Catalyst Performance in Ethanol Steam Reforming by Operando DRIFT Studies and DFT Modelling

Gabriela Grzybek[a], Olga Wasiłek[a], Magdalena Greluk[b], Grzegorz Słowik[b], Arantxa Davó-Quiñonero[c], Agustín Bueno-López[c], Dolores Lozano-Castelló[c], Paweł Stelmachowski[a], Filip Zasada[a], Witold Piskorz[a], and Andrzej Kotarba[a]*

*Corresponding author: g.grzybek@uj.edu.pl

[a]Faculty of Chemistry, Jagiellonian University in Krakow, Gronostajowa 2, 30-387 Krakow, Poland

[b]Faculty of Chemistry, Maria Curie-Sklodowska University; Maria Curie-Sklodowska Sq. 3, 20-031 Lublin, Poland

[c]Department of Inorganic Chemistry, University of Alicante Carretera de San Vicente s/n, E03080 Alicante, Spain

DFT MODELING

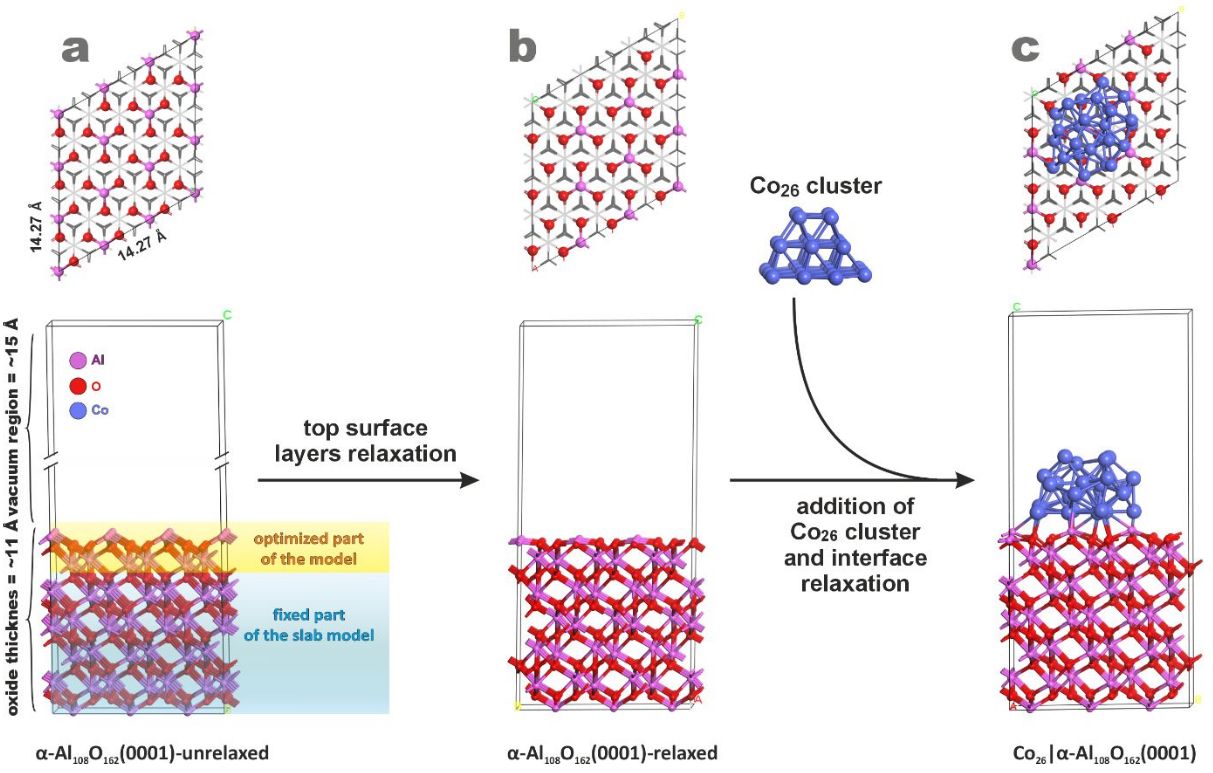

Figure S1. Slab models representing bare Al-terminated (0001) surface of α-Al_2O_3 support before (**a**) and after (**b**) geometry optimization, together with the slab model of Co_{26} cluster stabilized on the α-Al_2O_3(0001) plane and optimized (**c**).

The three-layer Co_{26} cluster (cut from bulk Co) with four Co atoms in the top layer, eight in the middle, and fourteen in the bottom was placed on the α-Al_2O_3(0001) plane and optimized (**Figure S1c**). During optimization a noticeable shift in the positions of surface atoms was observed, disrupting the initial bulk-like positions. The atoms of the lower layer adjust to the exposed oxide anions, forming several bonds of approximately ~2.2 Å in length, whereas the initial equivalent Co-Co bonds (2.47 Å in bulk Co) were disturbed (ranging from 2.12 to 2.44 Å). The top view of the relaxed Co_{26}|α-$Al_{108}O_{162}$(0001) model (top panel in **Figure S1c**) reveals that the chosen (3×3×1) dimension is large enough to accommodate the Co_{26} cluster without significant interaction with its periodically repeated images (the shortest distance between such clusters images is 6.7 Å).

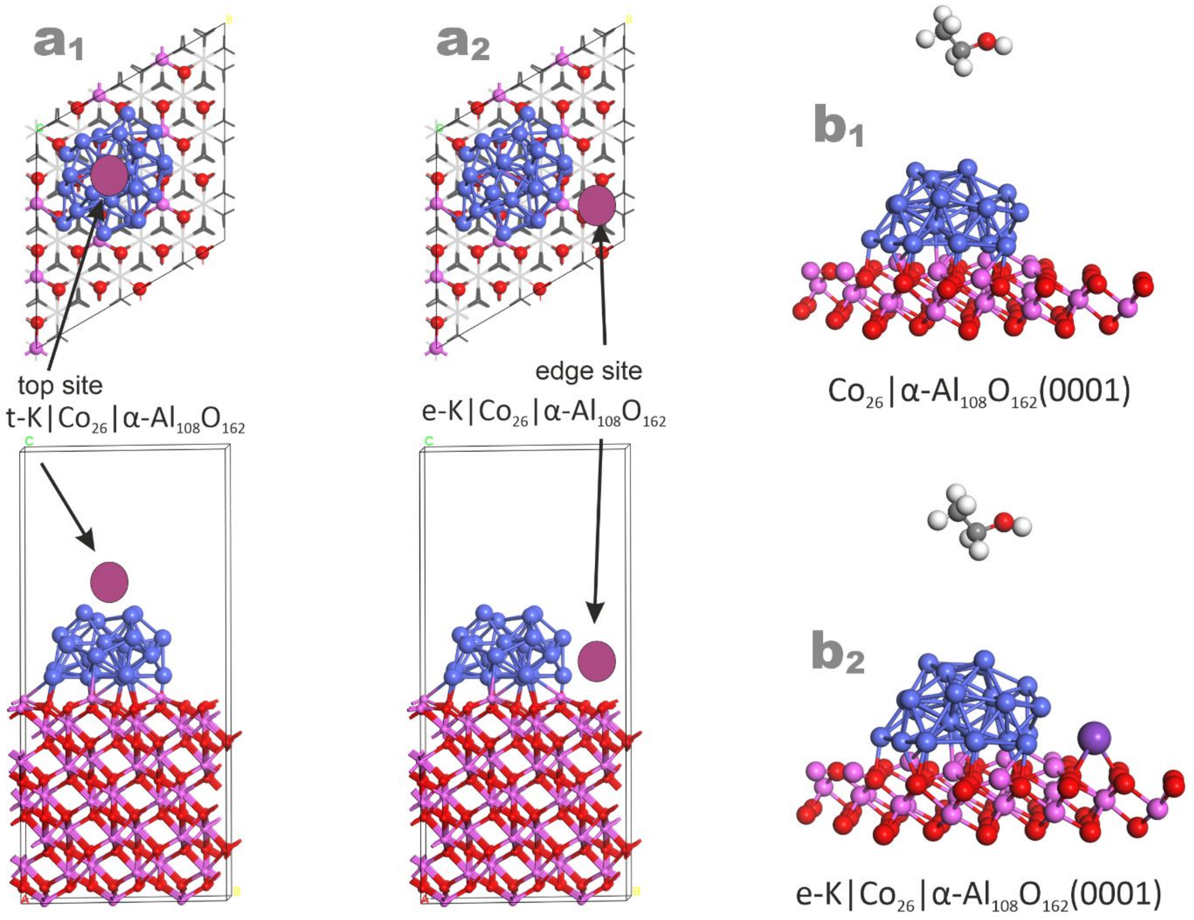

Figure S2. The top and side views of the optimized slab model representing Co_{26} cluster attached to the α-Al_2O_3(0001) surface together with potassium ion placed in top (a_1) and (a_2). Optimized part of the slab models used for modelling of the key steps of the ESR reaction over bare and potassium doped catalyst.

To determine the influence of potassium on the ESR reaction, the stabilization of K atom positioned on the top of the cobalt cluster (t-K|Co_{26}|α-$Al_{108}O_{162}$(0001) **Figure S2a$_1$**) and on its edge (e-K|Co_{26}|α-$Al_{108}O_{162}$(0001) **Figure S2a$_2$**) were compared. The edge potassium was found to be more stable (by 1.44 eV) and such a model was selected for further investigation. Finally, the developed Co_{26}|α-$Al_{108}O_{162}$(0001) and e-K|Co_{26}|α-$Al_{108}O_{162}$(0001) models were modified by the addition of ethanol molecule (**Figure 2b$_1$** and **b$_2$**), and computational experiments regarding CH_3CH_2OH reactivity in contact with cobalt centers in the presence and absence of potassium were conducted.

Partial charges distribution within the cobalt cluster

The partial charges distribution within the Co_{26} cluster on the α-Al_2O_3 (0001) surface without and with potassium dopant is listed in **Table S1**. In the first case, only a small charge transfer(of -0.08 |e| onto the cobalt cluster is expected (see bottom row of Table S1) and bottom cobalt atoms acquire slightly more negative charge than top ones. However, for the potassium-doped surface, the charge flow is much more pronounced (–1.126 |e|). In this case, the top and middle layer Co atoms are almost metallic (with the most charges in the range of 0.01–0.11 |e|) and may be denoted as Co^0 sites whereas bottom layer Co atoms have gained slightly negative charge (q_B is in the range of -0.056 to -0.254 |e|) and may be treated as partially reduced Co^{x-} sites. A similar effect (of negatively charged Co clusters over potassium-doped Co|Al_2O_3 catalyst) was recently reported by Wang and coworkers (*Catal. Sci. Technol.,2017, 7, 3613-3625*).

Table S1. The charge distribution within the Co_{26} cluster on bare and potassium doped α-Al_2O_3 (0001) surface.

Layer	Co	Bader charge (q_B) / |e|	
		Co_{26}|α-$Al_{108}O_{162}$	K|Co_{26}|α-$Al_{108}O_{162}$
Top	1	0.021	0.052
	2	0.089	0.057
	3	0.037	0.096
	4	0.094	0.104
Middle	5	0.011	0.044
	6	0.039	0.011
	7	0.054	0.078
	8	0.059	-0.010
	9	0.001	0.080
	10	-0.055	-0.034
	11	-0.075	-0.050
	12	-0.030	0.042
Bottom	13	-0.025	-0.056
	14	0.019	0.011
	15	0.044	0.006
	16	-0.038	-0.038
	17	0.062	-0.108
	18	0.002	0.056
	19	-0.026	0.173
	20	-0.055	-0.116
	21	-0.083	-0.031
	22	-0.004	-0.044
	23	-0.043	-0.210
	24	-0.058	-0.191
	25	-0.072	-0.254
	26	-0.048	-0.182
All layers	SUM	–0.081	–1.126

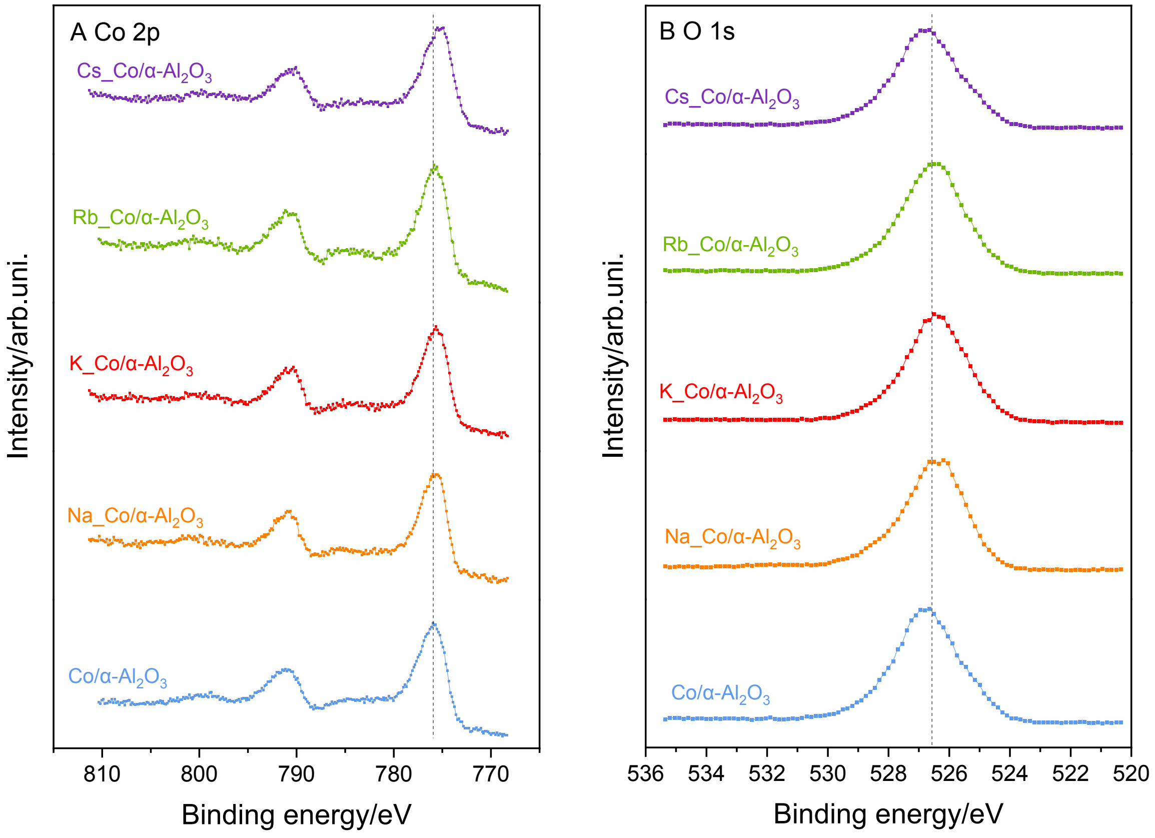

Figure S3. XPS spectra of Co 2p (A) and O 1s (B) core levels of the fresh Co/α-Al_2O_3 and alkali-doped Co/α-Al_2O_3 catalysts.

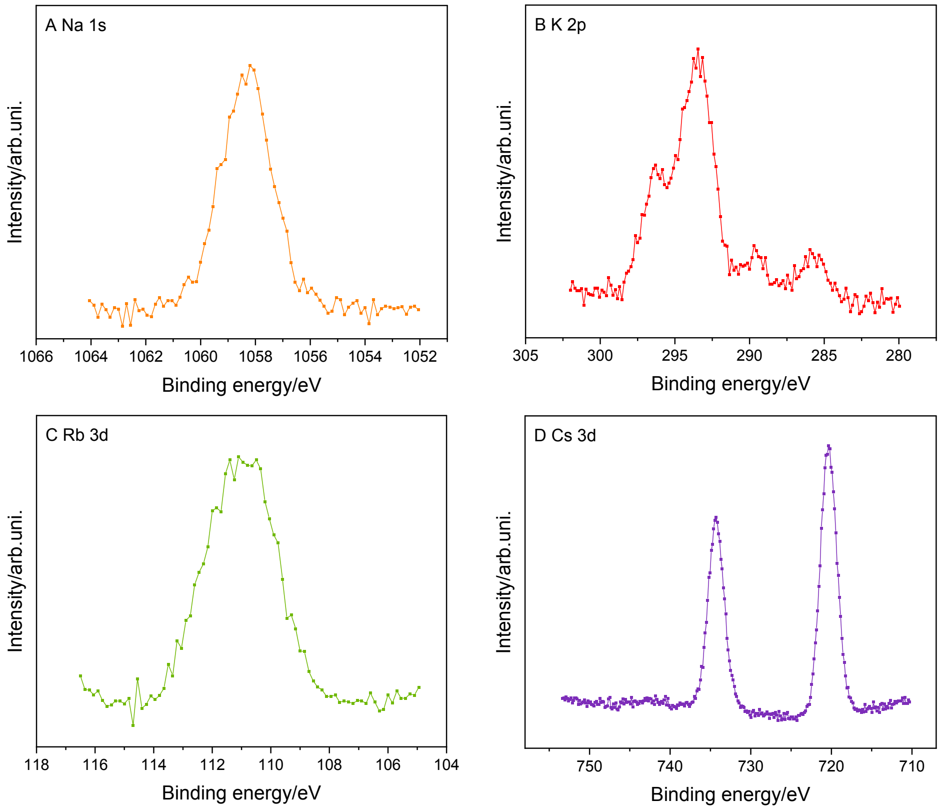

Figure S4. XPS spectra of Na 1s (A), K 2p (B), Rb 3d (C), and Cs 3d (D) core levels of the fresh alkali-doped Co/α-Al_2O_3 catalysts.

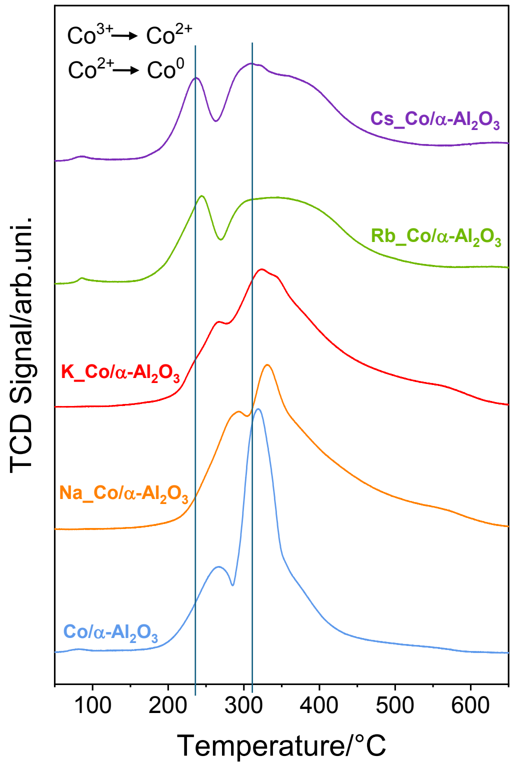

Figure S5. TPR profiles of the Co/α-Al_2O_3 and alkali-doped Co/α-Al_2O_3 catalysts.

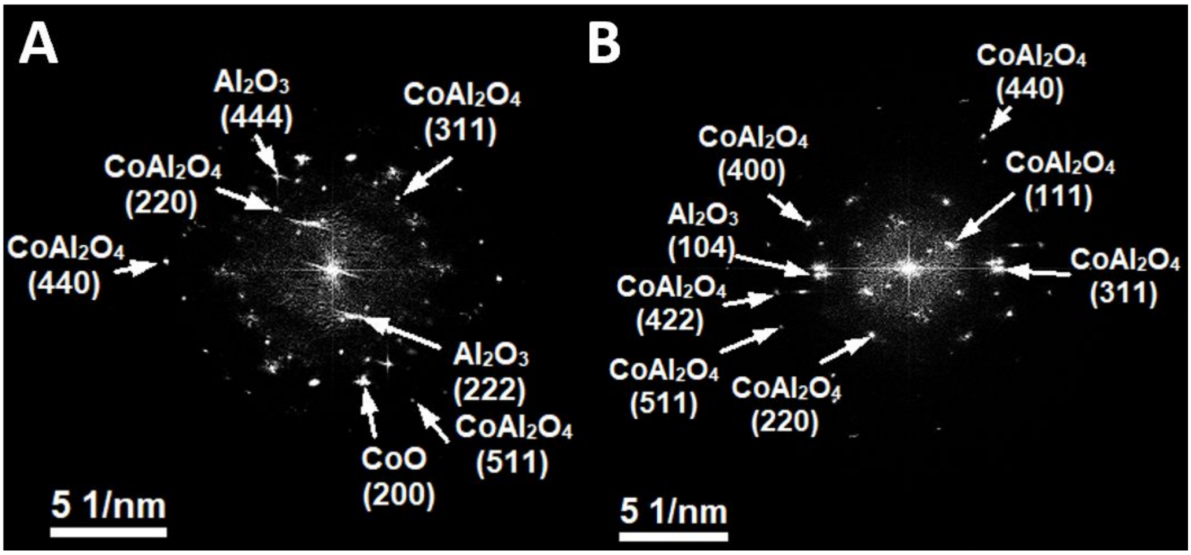

Figure S6. The Fast Fourier Transform (FFT) patterns extracted from HREM images for: A - Co/α-Al_2O_3 A) and B) K_Co/α-Al_2O_3 samples

Identification of the phases on the surface of the catalysts was conducted. The determined interplanar distances for the Co/α-Al_2O_3 sample (Figure S4A) were 2.28, 1.43, 1.56, 2.86, 2.45, 2.12, and 4.60 Å, corresponding to the lattice planes (444), (440), (551), (220), (311), (200), and (222), respectively. For the potassium-doped K_Co/α-Al_2O_3 sample (Figure S4B) the distances 4.67, 1.56, 2.86, 1.66, 1.43, 2.45, and 2.02 Å corresponded to the lattice planes (111), (511), (220), (422), (440), (311), and (400) of the $CoAl_2O_3$ spinel. These results were confirmed by complementary XRD and Raman spectroscopy techniques (Figure 1).

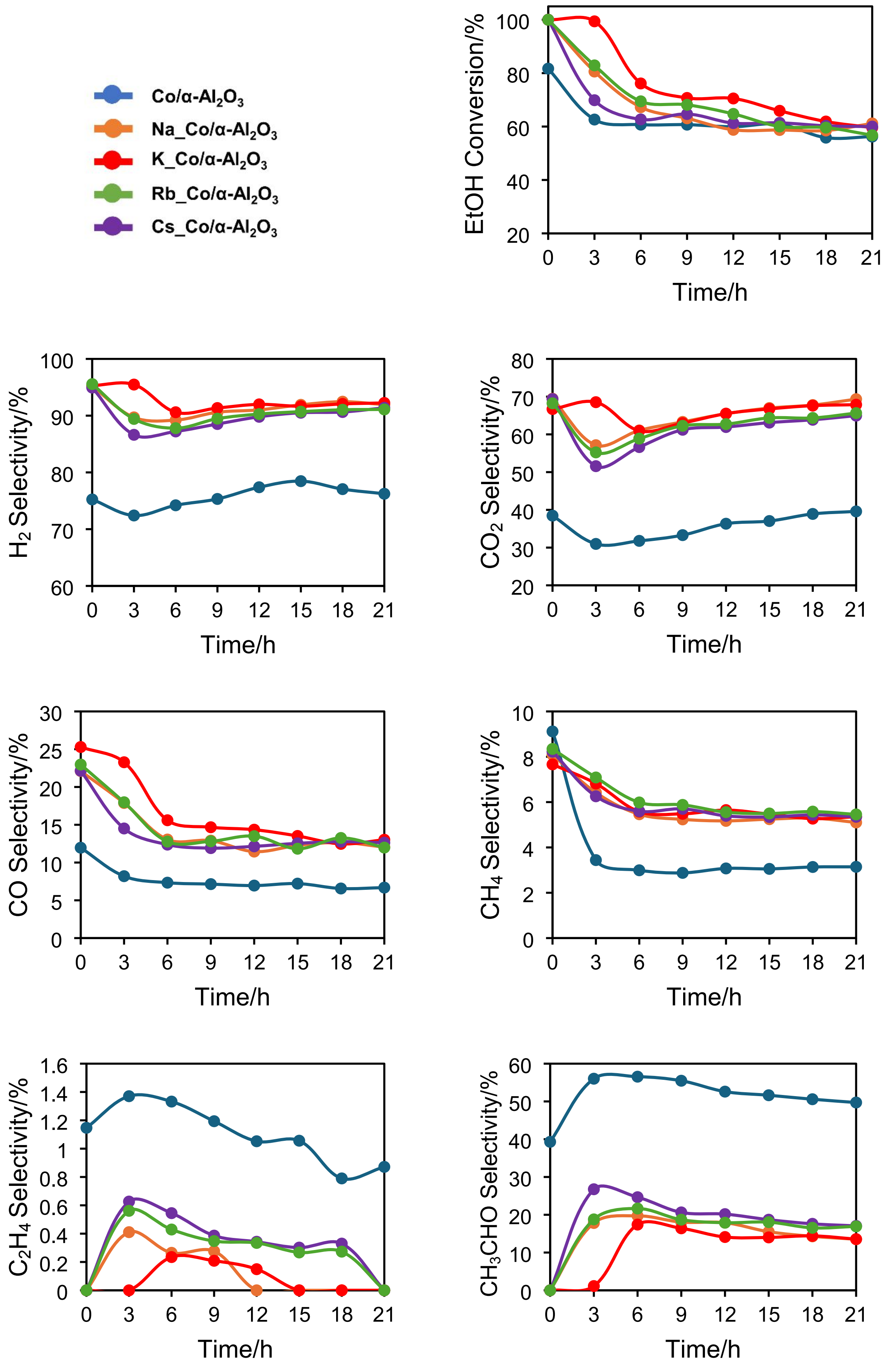
Co/α-Al2O3
Na_Co/α-Al2O3
K_Co/α-Al2O3
Rb_Co/α-Al2O3
Cs_Co/α-Al2O3
EtOH Conversion/%
H2 Selectivity/%
CO2 Selectivity/%
CO Selectivity/%
CH4 Selectivity/%
C2H4 Selectivity/%
CH3CHO Selectivity/%
Time/h

Figure S7. Results of catalytic tests: ethanol conversion and selectivity to ESR reaction products for the Co/α-Al_2O_3 and alkali-doped Co/α-Al_2O_3 catalysts.

Table S2. Assignment of bands by species present in the ESR reaction observed by DRIFT

Wavenumber Co/α-Al_2O_3	Wavenumber K_Co/α-Al_2O_3	Vibrational mode	Species
3739	3733	$\upsilon(OH)$	OH – hidroxile – typ I
3666	3665	$\upsilon(OH)$	OH – hidroxile – typ II
2968	2970	$\nu_{as}(CH_3)$	CH_3CH_2O ethoxy
2904	2906	$\nu_s(CH_3)$	CH_3CH_2O ethoxy
2718	2702	$\nu_s(CH_3)$	CH_3CH_2O acetaldehyde
2356	2358	$\nu_{as}(OCO)$	CO_2
2310	2310	$\nu_s(OCO)$	CO_2
2178	2180	$\upsilon(CO)$	CO
2109	2160	$\upsilon(CO)$	CO
1746	1746	$\upsilon(C=O)$	acetaldehyde
1573	1586	$\nu_{as}(OCO)$	CH_3CO_2 - acetate
1508	1507	$\delta_{as}(CH_3)$	CH_3CO_2 - acetate
1452	1453	$\nu_s(OCO)$	CH_3CO_2 - acetate
1394	1396	$\upsilon(CO)$	CO_3 – polidenetate carbonate
1377		$\delta_s(CH_3)$	CH_3CH_2O ethoxy
1336	1339	$\delta_s(CH_3)$	CH_3CO_2 – acetate/ CO_3 – polidenetate carbonate
1314	1314	CH_4 deformation	methane
1250	1250	δ_{as} (CH_3)	Hydrogen carbonate
1224	1226	$\upsilon(CO)$	Acetyl
1078	1073	$\upsilon(CO)$ Bi (type II)	CH_3CH_2O ethoxy
1048	1048	$\upsilon(CO)$ Bi (type II)	CH_3CH_2O ethoxy/CO
978	974	$\eta2$ $\rho(CH_3)$	CH_3CH_2O acetaldehyde

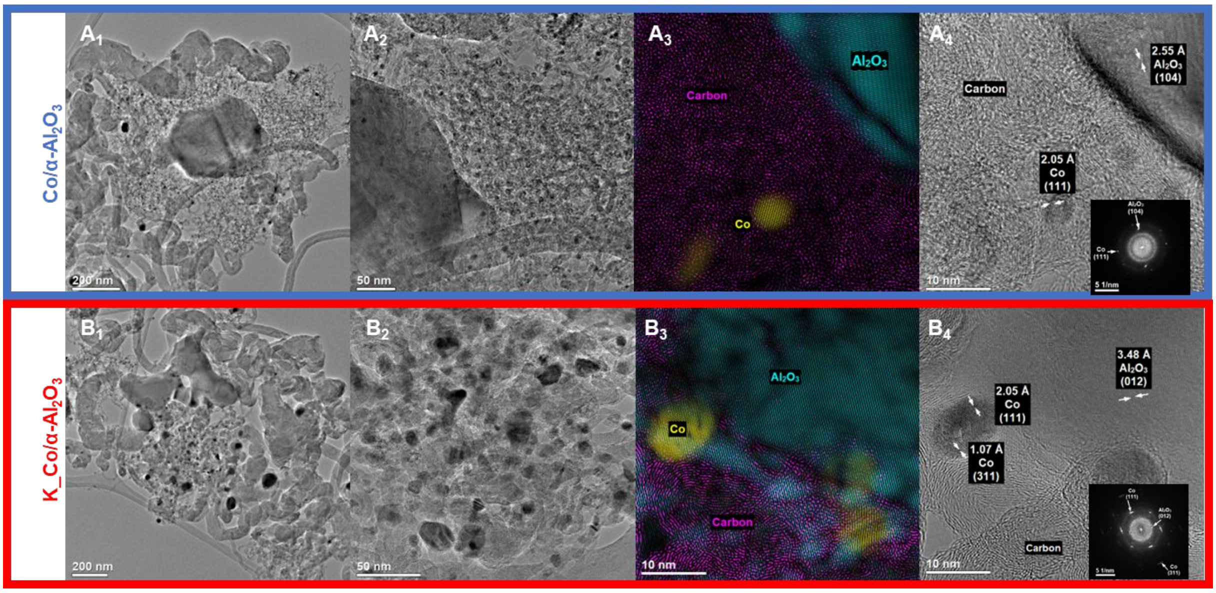

Figure S8. Microscopic analysis of the A) Co/α-Al_2O_3 and B) K_Co/α-Al_2O_3 catalysts after ESR (TEM images and phase identification).

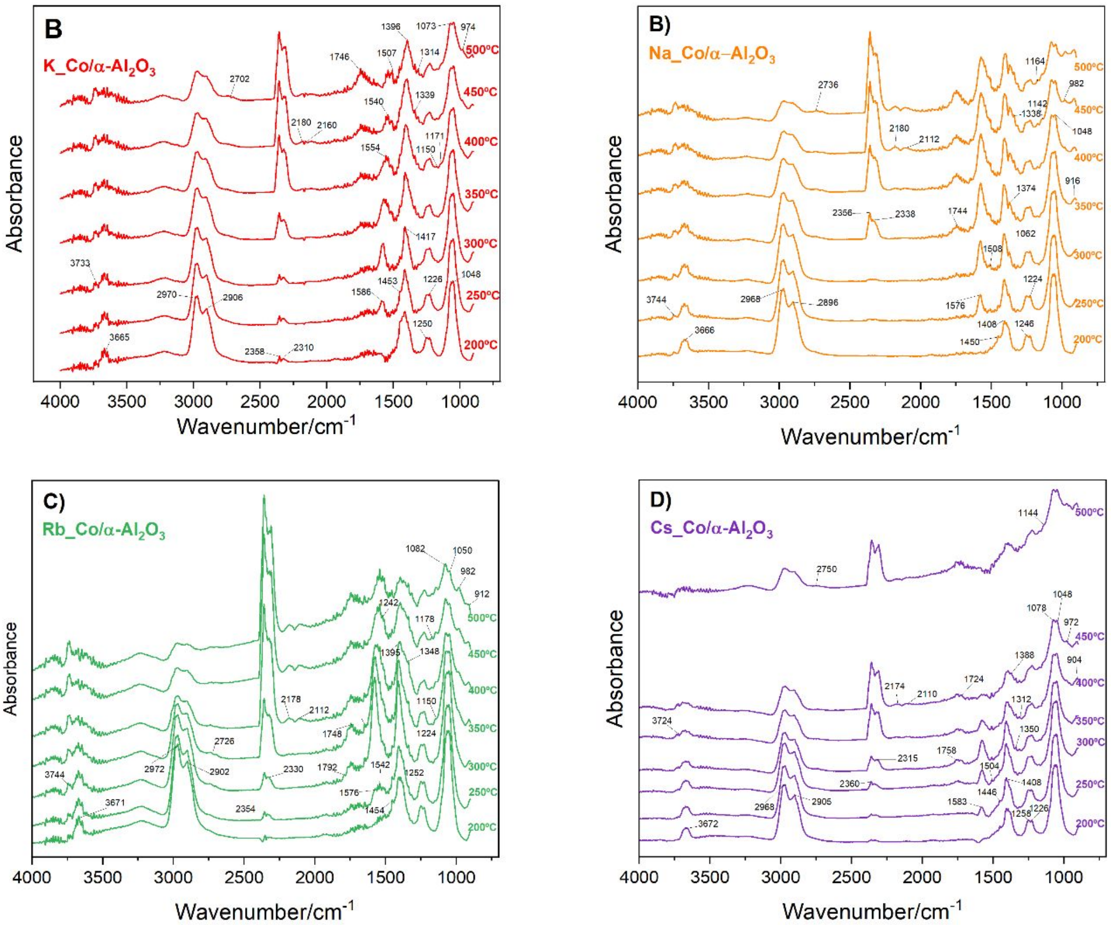

Figure S9. DRIFT spectra of the Co/α-Al_2O_3 and alkali-doped Co/α-Al_2O_3 catalysts under EtOH:H_2O reaction mixture conditions (1:4)
